# Healthy Eating for Successful Living in Older Adults™ community education program—evaluation of lifestyle behaviors: A randomized controlled trial

**DOI:** 10.3389/fragi.2022.960986

**Published:** 2022-09-06

**Authors:** Junaidah B. Barnett, Wu Zeng

**Affiliations:** ^1^ Health and Healing Research Education and Service, Boston, MA, United States; ^2^ Department of Nutrition, Harvard School of Public Health, Harvard University, Boston, MA, United States; ^3^ Tufts University Sensory and Science Center, Medford, MA, United States; ^4^ Department of Global Health, School of Health, Georgetown University, Washington, DC, United States; ^5^ Schneider Institutes for Health Policy, The Heller School for Social Policy and Management, Brandeis University, Waltham, MA, United States

**Keywords:** Healthy Eating for Successful Living in Older Adults^™^, HESl, older adults, lifestyle behaviors, evaluation, HESL community education program

## Abstract

**Objective:** Older adults face many chronic health issues including heart disease and osteoporosis, which are preventable through changes in lifestyle behaviors. The Healthy Eating for Successful Living in Older Adults™ (HESL) is a 6-week community education program designed specifically for persons aged ≥60 years, to promote behavioral changes toward a healthy lifestyle. Our objective is to evaluate the HESL. This is the first official evaluation of the HESL since its initiation in 2005.

**Study Design:** A cluster randomized controlled trial.

**Method:** Program implementation and evaluation took place between July 2018 and January 2020. Twenty-nine sites, with 292 participants aged ≥60 years from across five states (mostly from Massachusetts), were randomized into the intervention group (IG) (16 sites; *n* = 150 participants) and control group (CG) (13 sites; *n* = 142 participants). The HESL workshops followed a scripted curriculum including information from the USDA’s MyPlate™ and the USDA 2015–2020 dietary guidelines. Intervention elements included goal setting, self-assessment, group support, and problem solving through brainstorming. The CG received no intervention. Outcome measures were collected in both groups at baseline, 2 weeks postintervention (week 8), and 6 months postintervention. These included self-reported lifestyle behaviors, a composite healthy behavior index (HBI), body mass index [weight (kg)/height (m^2^)], and waist-to-hip circumference ratio (WHR). Mixed-effects regression models were used to examine the impact of the intervention.

**Results:** The IG showed significantly improved responses to most healthy lifestyle behavior questions at week 8 compared to the CG. However, not all improved responses were sustained at month 6. Significant improvements detected at month 6 included responses to the question on making food choices that are healthy for the heart, using MyPlate™ tools for food choices, reading nutrition labels when shopping/planning meals, and confidence in managing own health (*p* < 0.001 in most cases). HBI was significantly improved at week 8 and month 6 (*p* < 0.001). WHR decreased significantly (*p* < 0.05) at month 6.

**Conclusion:** Positive changes in lifestyle behaviors and WHR were observed in older adults due to the HESL intervention.

**Clinical Trial Registration:** clinicaltrial.gov, Identifier: NCT04991844; https://clinicaltrials.gov/ct2/show/NCT04991844

## Introduction

Greater than 60% of adults over the age of 65 have been reported to have more than one chronic condition (Ward and Schiller, 2013), including heart disease, cancer, stroke, diabetes, osteoporosis, obesity, and Alzheimer’s disease (Kirkman et al., 2012; Halter et al., 2014; Mark Mather and KelvinPollard, 2015; Arauco Lozada et al., 2021; Fahimfar et al., 2021; Falaschi et al., 2021); these diseases have devastating effects on their functional capability and quality of life. By 2049, functional disability, such as hip fractures and stroke, due to chronic diseases in older persons**,** is expected to increase at least 300 percent (Boult et al., 1996). Severe, immediate, and progressive disabilities lead to the inability of older adults to care for themselves (Fried and Guralnik, 1997). In addition, health care costs for older adults with chronic diseases and functional impairments, including home care expenses, are high and continue to grow (Stuck et al., 2004; Medical Spending of the Elderly, 2015). The burden of various chronic diseases such as heart disease and osteoporosis can be reduced with changes in lifestyle behaviors. Changes in lifestyle behaviors such as making healthy food choices, increasing physical activity, improving sleep quality, smoking cessation, and maintaining a healthy body weight have been shown to help prevent, slow, stop, or even reverse various chronic diseases (Barnard et al., 1994; Ornish et al., 1998; Hu and Willett, 2002; Roberts and Barnard, 2005; Ornish et al., 2008; Barnard et al., 2009; Frates, 2016; Orenstein et al., 2016; Benjamin et al., 2017) and to increase life expectancy (Loef and Walach, 2012), with great potential to improve quality of life and reduce health care costs. It is therefore important to help empower older adults toward self-care and well-being. This can be accomplished with the development of effective evidence-based interventions that are safe, relatively low-cost, and scalable and have the most potential for the largest impact (Frieden, 2014).

To address this need, in 2005, Hebrew SeniorLife and its associated partners designed and piloted a new program for older adults focused on nutrition and healthy lifestyle behaviors. The program—Healthy Eating for Successful Living in Older Adults™ (HESL)—provides older adults with needed knowledge on healthy food choices and lifestyle behaviors and tools that support behavioral changes (details of the HESL intervention are described below). At present, HESL serves more than 1,000 older persons yearly. The program is available in English, Spanish, and Russian, with plans to translate the intervention for Chinese and Portuguese speakers. The program is now disseminated through its training center at AgeSpan, previously known as the Elder Services of the Merrimack Valley and North Shore, located in Lawrence, Massachusetts. AgeSpan licenses and trains councils on aging, senior centers, congregate housing sites, neighborhood health centers, community centers, faith-based organizations, and others to deliver the program to the older persons in their communities. The program, available in both in-person and remote delivery models, is currently being offered in all 14 counties including 50% of cities and towns in Massachusetts, as well as in 13 additional states.

This study presents data on an evaluation of the HESL and examines the impact of the intervention on factors such as healthy behaviors, food choices, and quality of life at 2 weeks post-intervention (week 8) and 6 months post-intervention. This is the first official evaluation of the HESL since its initiation in 2005.

## Methods

### Study design

A cluster unblinded randomized controlled trial in persons aged ≥60 years, recruited from community-based settings, was conducted between July 2018 and January 2020. The intervention group (IG) was compared with the control group (CG) receiving no intervention to evaluate the effects of the 6-week HESL intervention on outcome measures of interest at week 8 (i.e., at 2 weeks post-intervention), and at 6 months post-intervention. These time points were selected because of interest to determine the short-term or more immediate impact of the intervention, as well as the longer-term impact of 6 months post-intervention.

**FIGURE 1:**
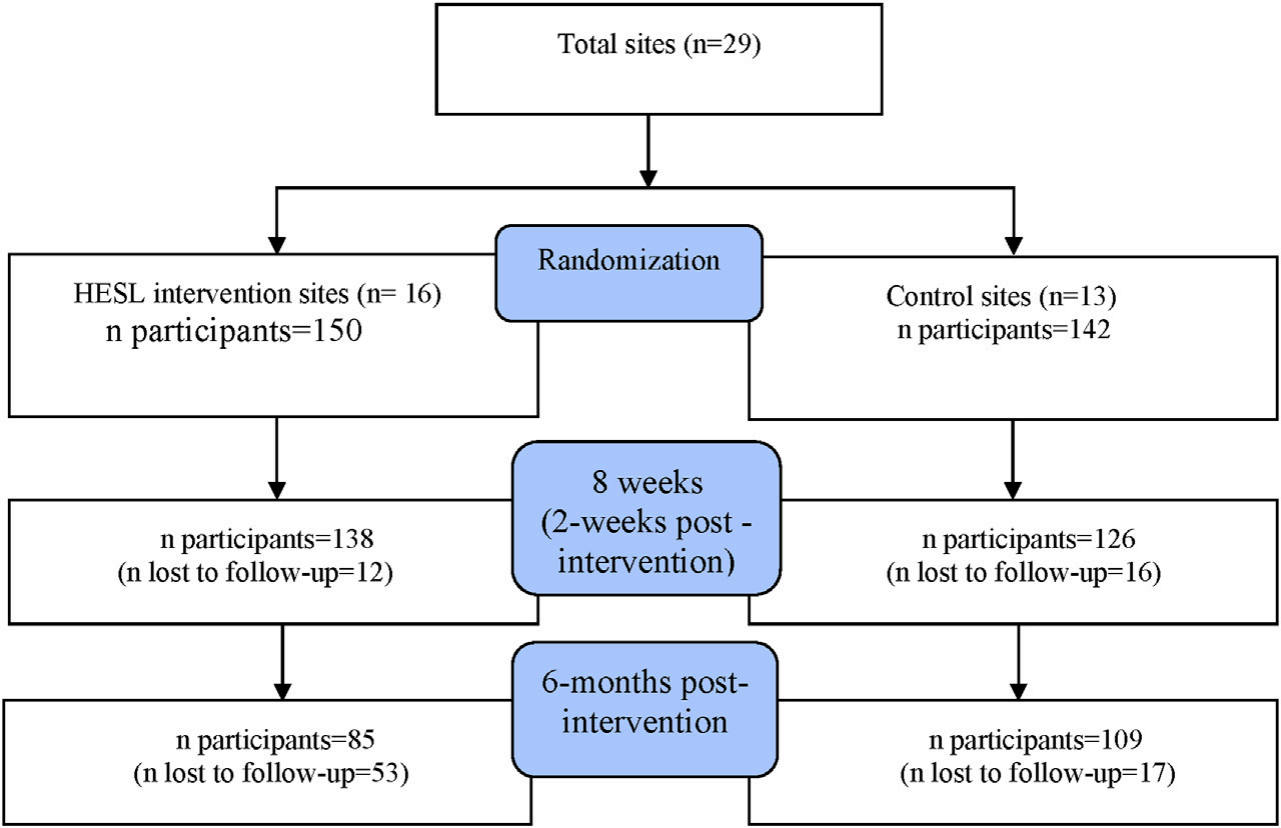
Study Consort Diagram.

A biostatistician who was independent of the program implementers conducted the randomization at the site level [i.e., senior centers (*n* = 14), housing authorities (*n* = 4), churches (*n* = 3), assisted living facilities (*n* = 6), and library (*n* = 2)]. A total of 29 sites across five states (Massachusetts, Maryland, Florida, Rhode Island, and Michigan) were randomly assigned to the HESL IG and CG, using a single randomization approach with computerized random numbers. Sixteen sites were assigned to the IG, and 13, to the CG. All participants recruited in the IG sites were assigned as IG participants, and those recruited in the CG sites were assigned CG participants by the program implementers. Figure 1 shows the consort diagram of the study. The sample size was determined based on the assumption that the intervention would increase the mean fiber intake (g/day) by 30%, an indicator for a parallel study. With a type I error of 5% and a type II error of 80%, as well as a 25% loss of follow-up, we estimated a sample size of 125 per group.

Participant inclusion criteria included: 1) male or female aged ≥60 years, 2) English speaking, and 3) willing to participate and complete all study activities following randomization into the IG or CG. Those eligible, interested, and able to participate were recruited into the study.

Both IG and CG met at three specified time points, i.e., at baseline, week 8, and month 6, for the completion of study questionnaires and to provide anthropometric measures. In addition, the IG met weekly to receive the intervention outlined below.

The protocol was approved by the New England Institutional Review Board, and written informed consent was obtained from all participants. This study was registered at clinicaltrial.gov with identifier: NCT04991844. https://clinicaltrials.gov/ct2/show/NCT04991844.

### Healthy Eating for Successful Living in Older Adults™

The HESL intervention was developed with strategies aimed to promote changes in lifestyle factors in adults aged ≥60 years.

The IG met once a week, for 2.5 h, with 10–16 participants/group, over a period of 6 weeks for the HES workshops. The HES workshops, offered in English, followed a scripted curriculum that incorporated information from the USDA’s MyPlate™, and the USDA 2015–2020 dietary guidelines (U.S. Department of Health and Human Services and U.S. Department of Agriculture, 2015). The HESL was designed to increase knowledge of healthy eating habits and identify food choices for healthy bones and heart, and for overall health. Participants were taught how to select healthier foods using the MyPlate™ app and to read labels, plan menus, and prepare meals in a lively interactive session of learning as a group. Among others, MyPlate™ emphasizes the importance of eating a variety of foods, eating foods from five major food groups (fruits, vegetables, grains, protein, and dairy), covering half their plates with fruits and vegetables, making half of the grain intake whole grains, choosing low or no fat dairy products, choosing foods with less added sugar and salt, avoiding transfat and saturated fat, reducing intake of cholesterol, eating more fiber, and being physically active. MyPlate™ is also individualized for each person based on his/her age, gender, and level of physical activity. Participants were thus guided on how to determine their caloric requirements and what to eat.

Participants were also taught goal setting, problem solving through brainstorming, group support, self-assessment, and management of dietary and physical activity patterns. At the end of every session, each participant was asked to set a goal for the new week related to healthy eating. The goals were focused on a nutrition challenge the participant would like to address and felt confident was achievable before the next week’s session. Participants were taught to formulate their own SMART goals, i.e., goals that are specific, measurable, action-oriented, realistic, and time-sensitive, which incorporate accountability and monitoring (Frates et al., 2019). Participants kept a food and physical activity journal to monitor changes and identify problem areas in their eating habits and physical activity. By monitoring food choices, and physical activity type and intensity through the completion of a food and physical activity journal, participants were in a position to identify healthy changes made in their dietary and physical activity patterns as well as identify barriers to reaching goals. This self-assessment component is an important factor of the intervention.

These workshops were led by program leaders who were trained and certified to present materials and support brainstorming to solve a problem or overcome barriers to success, identified by participants, using culturally relevant solutions, as well as the promotion of socialization and group interaction. These are also important components of the intervention. In addition, participants were given supporting materials such as a “Participant Manual” with clear dietary intake and physical activity guidelines. Participants were also provided with information on the availability of community nutrition and health education resources. The intervention also included various “hands-on” activities such as visiting a grocery store, learning to read food labels, and making healthy choices in preparing meals and at a restaurant. A Registered Dietitian/Nutritionist was available to help answer questions that remained unanswered by the workshop leaders, and responses were brought back to participants the following week. Table 1 provides a summary of the workshops’ weekly curricula.

**TABLE 1:** Summary of healthy eating for successful living workshops’ weekly curricula.

Week	HESL workshops’ weekly curricula
Week 1	Use of MyPlate™, and the 2015–2020 dietary guidelines as a foundation for healthy eating, label reading, identifying and overcoming barriers to healthy eating, and importance of maintaining a healthy weight and physical activity
Week 2	How to make healthy choices from the Grains, Vegetables, and Fruits Groups, understand the importance of Water and Nutrition, Facts on food labels, and how to incorporate endurance exercises
Week 3	How to make healthy choices from the Protein and Dairy Groups, benefits of plant-based proteins in preventing certain chronic diseases, such as coronary heart disease, diabetes, and colon cancer, how to limit sodium intake, fats that are good and bad for health, healthy food preparation and storage methods, importance of calcium and vitamin D for bone health, and sources of these vitamins (from both foods and supplements), and importance of balance exercises
Week 4	How to create a well-balanced diet that includes Fats, and Sweets, and understanding the roles of Fats and Sweets in disease prevention, including heart disease prevention, how to modify recipes for health, importance of strength exercise, and preparation for Week 5 and Week 6 activities
Week 5	Applying Grocery Shopping Skills, practice label reading skills, identifying and modifying shopping list to include more heart healthy and bone healthy food choices
Week 6	Putting it all together—planning menu and preparing heart and bone healthy foods using techniques that are low in fat and added calories following MyPlate™ and flexibility exercises

### Data collection

#### Demographic variables

Participant’s age, gender (male = 1, female = 0), race (seven categories; recoded as 1 for White; 0, otherwise), education (eight categories, recoded as 1 for those with a college degree and above; 0, otherwise), and marital status (eight categories; recoded as 1 for married or having domestic partnership; 0, otherwise). Information on household income was excluded as almost 80% of participants did not respond to this question.

#### Healthy behavior questions

These self-reported healthy behavior questions were formulated and used in-house to specifically evaluate the impact of the HESL intervention in the past. These questions and their transformations into binary variables are listed in Table 2. In addition, we generated a composite healthy behavior index (HBI) by summing the dichotomous items. HBI scores ranged between 0 and 10; a higher score indicates more healthy behaviors.

**TABLE 2:** Healthy lifestyle behavior questions (transformed to binary variables).

Healthy Lifestyle Behavior Questions	Original Responses	Transformed Binary Responses
Do you make food choices that are healthy for your bones?	Always; Most of the time; Sometimes; Rarely; Never	1 = always, most of the time, or sometimes; and 0 = rarely and never
Do you make food choices that are healthy for your heart?	Always; Most of the time; Sometimes; Rarely; Never	1 = always, most of the time, or sometimes; and 0 = rarely and never
Do you read nutrition labels when shopping or when planning meals?	Always; Most of the time; Sometimes; Rarely; Never	1 = always, most of the time, or sometimes; and 0 = rarely and never
Do you use MyPlate™ tools to help make food choices?	Always; Most of the time; Sometimes; Rarely; Never	1 = always, most of the time, or sometimes; and 0 = rarely and never
Are you currently a smoker?	Yes; No	1 = Yes; 0 = No
During the past month, how many hours of actual sleep did you get most nights?	<4 h; 4 h; 5 h; 6 h; 7 h; 8 h; 9 h; 10 h	1 = 6–8 h of sleep; 0 = ≤ 5 h and ≥ 9 h
During the past month, how would you rate your sleep quality overall?	Very good; Fairly good; Fairly bad; Very bad	1 = Very good or Fairly good; 0 = otherwise
How confident are you that you can manage most of your health problems	0–10; with 10 being highest confidence	1 = ≥ 6; 0 if ≤ 5
How understandable and useful is the information that your doctor or nurses have given you about your health problems or concerns	0–10, with 10 being most understandable and useful	1 = ≥ 6; 0 if ≤ 5
I play an active role in my health care and well-being	0–10, with 10 being most active	1 = ≥ 6; 0 if ≤ 5

#### Social connectedness

This was measured using the Berkman–Syme Social Connectedness Survey (Berkman and Syme, 1979), which contained 11 items, with questions such as “How many close friends do you have?” We generated a continuous score using the same approach as done by Salinas et al. (2017); a high score implies greater social connectedness. Evidence for the predictive validity of this self-reported survey is available in Berkman and Breslow (1983).

#### Physical activity

Using a physical activity questionnaire, participants reported their average amount of time spent per week (ranging from 0 min to ≥11 h) during the previous month doing various activities. Using the classification by Ainsworth et al. (1993), metabolic equivalent task (MET)-hours/week were estimated by multiplying the MET score for each activity by the reported hours per week and summing across all activities (Colditz et al., 2003; Eliassen et al., 2010). This self-reported physical activity measure is used extensively in research studies and has been validated in both men and women (Al-Shaar et al., 2022; Pernar et al., 2022).

#### Anthropometrics

Participants provided data on their height at baseline, weight, and waist and hip circumferences (WC and HC) measures at all time points. Body mass index (BMI, kg/m^2^) and waist-to-hip circumference ratio (WHR) were computed. Self-reported anthropometric measures such as weight, height, and WC and HC have been observed to be valid measures in men and women (Rimm et al., 1990; Hodge et al., 2020). In this study, the workshop leaders trained the study participants on how to take their anthropometric measures and were there to supervise and assist participants with taking their measurements. A weighing scale was made available to participants at the workshop to take their weights, and a tape measure was given to each participant to take both their WC and HC.

#### Quality of life

Quality of life was measured using the Euro-QoL-5D-5L questionnaire (Janssen et al., 2013). The questionnaire solicits responses on mobility, self-care, usual activities, pain/discomfort, and anxiety/depression. Participants provided a rating of their own health on a scale of 0–100, where 0 means “death” and 100 means “the best health.” This self-reported quality of life questionnaire is available in more than 150 languages and is used as a quantitative measure of health outcomes that reflects the patient’s own judgment (Group E, 2022).

### Statistical analyses

Descriptive analyses for all measures between the IG and CG at baseline, week 8, and month 6 were conducted using the student *t*-test for continuous variables or the chi-square test for categorical variables. The impact of the intervention at week 8 and month 6 without adjusting for covariates were estimated using a difference in differences (DID) approach (unadjusted DID) using the following linear regression model A:

$${\text{y}}_{\text{cit}}={\text{β}}_{0}+{\text{β}}_{1}\text{group}+{\text{β}}_{2}\text{week }8+{\text{β}}_{3\;}\text{month }6+{\text{β}}_{4}\text{group}*\text{week }8+{\text{β}}_{5}\text{group}*\text{month }6+{\text{ε}}_{\text{cit}}$$

where y_cit_ represents outcome measure of interest (continuous or binary variables) specified in the data collection section above, for individual i, at time t, in cluster (or site) c; t implies the three study time points; group is the variable indicating whether the individual belongs to the IG (group = 1) or CG (group = 0); week 8 and month 6 indicate whether the measure is at the respective time points; group*week 8 and group*month 6 are the interaction terms between group and these time points, respectively; and ${\text{ε}}_{\text{cit}}$ is random noise. The coefficients for group*week 8 and group*month 6 ( ${\text{β}}_{4}\;\text{and}\;{\text{β}}_{5}\;\text{respectively}$ ) represent the net impact of the intervention on the outcome measures of interest at the respective time points. For simplicity, we did not add the site effect and individual effect to the model.

The impact of the intervention at week 8 and month 6 adjusting for covariates and addressing repeated measures (adjusted DID) were estimated using linear mixed-effects regression models by adding covariates of $x_{it}$ (including age, gender, education, marital status, state, and race), individual random effects of $\alpha_{i}$ , and the site random effect of $\gamma_{c}$ to the above model A. Model B below is used for our adjusted analyses.

$${\text{y}}_{\text{cit}}={\text{β}}_{0}+{\text{β}}_{1}\text{group}+{\text{β}}_{2}\text{week }8+{\text{β}}_{3\;}\text{month }6+{\text{β}}_{4}\text{group}*\text{week }8+{\text{β}}_{5}\text{group}*\text{month }6\;+{\text{ β}}_{6}x_{it}+\alpha_{i}+\gamma_{c}+\;{\text{ε}}_{\text{cit}}$$

By including $\gamma_{c}$ in the model, we addressed the effect of the clustering of individuals within a site in the analysis.

Although our outcome measures contain binary variables, we opted to use linear models for both DID models to ease the interpretation of our findings. However, we also presented the results from logit mixed-effects models (Rich-Edwards et al., 2019) for binary variables in Supplementary Table S1, which shows that there were no major differences in findings between the linear mixed-effects and logit mixed-effects models in terms of the significance of coefficients and the direction of the intervention impact. In addition, we presented relevant results between groups for participants who provided data at all three time points in Supplementary Tables S2,S3. All the analyses were conducted with Stata 16.0 software (STATA Corp, College Station, TX; Stata, RRID:SCR_012,763).

## Results

Table 3 shows the baseline characteristics of the participants (*N* = 292) in the CG (*N* = 142) and IG (*N* = 150) (Figure 1). Most participants lived in Massachusetts. There were no statistical differences in age, marital status, and gender between groups. However, the IG had significantly more White participants compared with the CG (*p* < 0.001). The IG also had more participants with some college education or above than the CG (*p* < 0.05).

**TABLE 3:** Characteristics of participants at baseline.

Characteristics	Control (*N* = 142)	Intervention (*N* = 150)	T Value or Chi-Square
Age (Mean ± SD)	72.7 ± 8.0	72.8 ± 10.2	−0.03
Female (%)	82.3	82.2	0.002
White (%)	59.3	77.2	11.11∗∗∗
Some college or above (%)	58.5	72.0	6.04∗
Married (%)	38.2	28.8	1.48
Location (% in MA)	83.8	76.1	2.70

∗*p* < 0.05; ∗∗∗*p* < 0.001. *p*-values obtained using student *t*-test for continuous variables, or chi-square test for categorical variables.

Findings from the unadjusted linear regression model A are presented in Table 4. Compared to findings at week 8, most improvements in the unadjusted values were sustained at month 6, except for making food choices for healthy bones. Significantly more participants in the IG compared with the CG indicated that they read nutrition labels at 6 months (*p* < 0.001).

**TABLE 4:** Intervention impact on lifestyle behaviors (all participants).

Evaluation questions (outcomes measures)	Control			Intervention			Unadjusted DID ^α^		Adjusted DID ^β^	
	Baseline	Week 8	Month 6	Baseline	Week 8	Month 6	Week 8	Month 6	Week 8	Month 6
	*N* = 134	*N* = 119	*N* = 100	*N* = 139	*N* = 136	*N* = 82				
Made food choices for healthier bones (%)	49.3	50.4	58.0	55.4	77.9	68.3	21.4∗	4.14	24.0∗∗∗	7.8
Made food choices for healthier heart (%)	54.7	52.5	55.9	56.9	86.2	81.7	31.6∗∗∗	23.7∗∗	32.6∗∗∗	29.4∗∗∗
Read nutrition labels when shopping/planning meals (%)	39.3	48.0	52.3	53.0	73.0	84.5	11.3	18.5∗	12.0∗	21.7∗∗∗
Used MyPlate™ tools for food choices (%)	11.5	13.5	15.1	23.6	53.7	53.0	28.1∗∗∗	25.9∗∗	33.9∗∗∗	29.0∗∗∗
Current smoker (%)	2.7	4.0	2.8	5.2	3.7	2.4	−2.7	−2.79	−3.4	−1.4
Sleep hours in past month										
6–8 h (%)	77.8	78.4	77.6	76.0	75.2	80.7	−1.4	4.93	−3.5	5.2
Overall sleep quality in the past month										
Fairly good or very good (%)	75.9	72.6	75.7	79.5	85.5	86.4	9.3	7.11	9.9∗	8.9
Confidence in managing own health										
Score 6 to 10 (%)	90.9	91.9	88.7	76.5	91.2	92.8	13.7∗	18.5∗∗∗	16.5∗∗∗	21.1∗∗∗
Found information provided by health care providers useful/understandable										
Score 6 to 10 (%)	84.3	85.8	86.8	81.9	86.7	89.9	3.2	5.5	6.2	5.0
Played active role in own health care and well-being										
Score 6 to 10 (%)	88.8	91.9	93.5	84.1	97.8	97.6	10.6∗	8.80	9.7∗	7.2
Healthy Behavior Index (HBI) (Mean ± SD)	5.7 ± 2.0	6.0 ± 1.9	6.0 ± 2.0	8.2 ± 2.4	7.3 ± 1.5	7.5 ± 1.4	0.8∗	0.9∗	1.2∗∗∗	1.3∗∗∗

∗*p* < 0.05, ∗∗*p* < 0.01, ∗∗∗*p* < 0.001.

^**α:**^ Unadjusted DID denotes the difference in differences, accounting for the baseline difference between the control and intervention groups. For example, the unadjusted DID result was calculated as $\left(y_{week\;8}^{intervention}-y_{week\;8}^{control}\right)-\left(y_{baseline}^{intervention}-y_{baseline}^{control}\right)$ for week 8, and positive and negative numbers under this column indicate the net increase or decrease in response (in percentage points) due to the intervention, respectively, at week 8; same at month 6 for all binary variables. For HBI, values are actual net increase or decrease due to the intervention. *p* -values were derived using a linear regression model.

^**β:**^ Adjusted DID (i.e., adjusted intervention effect) and *p*-values derived from a random effects regression model that accounts for site clustering and repeated measures, with binary outcomes measures (%) (1 = healthy behavior, 0 = otherwise; note exception: current smoker = 1, 0 = otherwise), adjusting for group (intervention = 1, control = 0); week 8 (week 8 = 1, 0 = otherwise); month 6 (month 6 = 1, 0 = otherwise); age (continuous variable); gender (male = 1, female = 0); education (college and above = 1, 0 = otherwise), marital status (married = 1 and 0 = otherwise), state (Massachusetts = 1 and 0 = otherwise), and race (White = 1 and 0 = otherwise). Positive and negative numbers under this column indicate the net increase or decrease in response (in percentage points) due to the intervention, respectively, at week 8, same at month 6 for all binary variables. For HBI, values are actual net increase or decrease due to the intervention.

The impact of the intervention controlling for age, gender, race, educational and marital status, as well as baseline differences, on health behaviors are also presented in Table 4. (adjusted DID using model B). In general, findings remain consistent compared with the results from the unadjusted analyses for both week 8 and month 6. However, in contrast to the unadjusted findings, reading nutrition labels when shopping/planning meals and having fairly good or very good sleep quality were shown to be significantly improved in the adjusted results at week 8 (*p* < 0.05 in both cases). Adjusted findings also showed that at week 8, the IG showed significantly improved responses to these questions: making food choices that are healthy for bones (*p* < 0.001) and heart (*p* < 0.001), using MyPlate™ (*p* < 0.001), confidence in managing own health (*p* < 0.001), and playing an active role in managing their own health care and well-being (*p* < 0.05). A similar pattern was observed at month 6, except for responses to making healthy food choices for bones and sleep quality, where significant differences were no longer detected between groups in the adjusted results. The degree to which participants understood and found useful the information provided by their doctors and nurses about their health problems and concerns, at week 8 and month 6, was also not found to be statistically significant between groups in the adjusted findings. The continuous composite HBI scores showed a significant improvement at week 8 (*p* < 0.001) and month 6 (*p* < 0.001).

We used a logit mixed-effects model to examine the impact of the intervention on the binary variables as a sensitivity analysis. In general, we found similar results in the logit mixed-effects model (Supplementary Table S1). Adjusted findings on participants who provided data at all three time points are presented in Supplementary Table S2.

Table 5 presents unadjusted and adjusted results of our analyses showing the effects of the intervention on BMI, WC, HC, WHR, physical activity level, social connectedness, and quality of life at week 8 and month 6. Similar to the results from the unadjusted analyses, the adjusted analyses results did not show a significant impact of intervention at week 8 nor at month 6, except for a significant decrease in WHR at month 6 (*p* < 0.05) in the IG, in comparison with the CG. Adjusted findings on participants who provided data at all three time points are presented in Supplementary Table S3.

**TABLE 5:** Intervention impact on body mass index, waist-to-hip circumference ratio, physical activity, social connectedness, and quality of life (all participants).

Outcome measures	Control (Mean ± SD)			Intervention (Mean ± SD)			Unadjusted DID ^α^		Adjusted DID ^β^	
	Baseline	Week 8	Month 6	Baseline	Week 8	Month 6	Week 8	Month 6	Week 8	Month 6
	*N* = 142	*N* = 121	*N* = 84	*N* = 137	*N* = 96	*N* = 59				
BMI kg/m^2^	28.0 ± 5.8	27.9 ± 5.7	28.2 ± 6.0	29.6 ± 6.0	29.2 ± 5.9	29.4 ± 6.4	−0.30	−0.44	−0.55	0.18
Waist Circumference (WC) (inches)	37.7 ± 5.7	37.9 ± 5.9	37.2 ± 5.7	39.7 ± 5.5	40.6 ± 5.0	39.6 ± 5.9	0.70	0.34	−0.15	−0.15
Hip Circumference (HC) (inches)	42.2 ± 5.5	41.7 ± 5.6	41.5 ± 5.2	43.7 ± 5.0	44.1 ± 5.0	44.8 ± 6.9	0.84	1.73	−0.19	1.36
Waist to Hip Circumference Ratio (WHR)	0.89 ± 0.08	0.91 ± 0.08	0.90 ± 0.07	0.91 ± 0.09	0.92 ± 0.08	0.89 ± 0.09	−0.003	−0.02	0.001	−0.03∗
Physical Activity (MET-hrs/week)	50.5 ± 55.2	52.5 ± 50.3	56.2 ± 48.4	52.8 ± 95.2	51.8 ± 86.3	40.9 ± 57.0	1.88	−16.6	1.91	−0.37
Social Connectedness Score	4.6 ± 3.1	4.7 ± 3.0	4.7 ± 3.2	5.2 ± 3.2	4.8 ± 3.2	4.8 ± 3.2	−0.37	−0.50	−0.58	−0.53
Quality of Life	80.3 ± 13.7	81.7 ± 11.9	80.8 ± 12.1	78.6 ± 16.5	80.8 ± 13.5	79.3 ± 17.7	0.81	0.17	0.91	0.14

∗*p* < 0.05.

^**α:**^ Unadjusted DID denotes the difference in differences, accounting for the baseline difference between the control and intervention groups. For example, the unadjusted DID result was calculated as $\left(y_{week\;8}^{intervention}-y_{week\;8}^{control}\right)-\left(y_{baseline}^{intervention}-y_{baseline}^{control}\right)$ for week 8, and positive and negative numbers under this column indicate the net increase or decrease in value due to the intervention, respectively at week 8; sme at month 6. *p* -values were derived using a linear regression model.

^**β:**^ Adjusted DID (i.e., adjusted intervention effect) and *p*-values derived from a random effects regression model that accounts for site clustering and repeated measures, with outcome measures as continuous variables, adjusting for group (intervention = 1, control = 0); week 8 (week 8 = 1, 0 = otherwise); month 6 (month 6 = 1, 0 = otherwise); age (continuous variable); gender (male = 1, female = 0); education (college and above = 1, 0 = otherwise), race (White = 1 and 0 = otherwise), state (Massachusetts = 1 and 0 = otherwise), and marital status (married = 1, 0 = otherwise). Positive and negative numbers under this column indicate the net increase or decrease in value due to the intervention, respectively at week 8; same at month 6.

## Discussion

The IG showed significantly improved responses to most healthy lifestyle behavior questions at week 8 compared with the CG. However, not all improved responses were sustained at month 6. Significant improvements detected at month 6 included responses to the question on making food choices that are healthy for the heart, using MyPlate™ tools for food choices, reading nutrition labels when shopping/planning meals, and confidence in managing own health. The HESL intervention was also associated with a statistically significant increase in the composite HBI at week 8 and month 6 and a significant decrease in WHR at month 6.

Various elements of the HESL intervention that contribute to its improvement in changing a healthy lifestyle include promoting changes in habits through small increments by setting goals for the week at the end of each session. As described, participants were taught to formulate their own SMART goals, i.e., goals that are specific, measurable, action-oriented, realistic, and time-sensitive, which incorporate accountability and monitoring (Frates et al., 2019). By monitoring food choices, as well as physical activity type and intensity through the completion of a food and physical activity journal, participants were in a position to identify changes made in their dietary and physical activity patterns as well as identify barriers to reaching goals. Brainstorming to solve problems or overcome barriers to success using culturally relevant solutions and the promotion of socialization and group interaction were also likely to be contributing factors. In addition, participants were given supporting materials such as a “Participant Manual” with clear dietary intake and physical activity guidelines. Participants were also provided with information on the availability of community nutrition and health education resources. Further, workshop leaders had access to a registered dietitian/nutritionist who helped answer questions raised by participants that remain unanswered during the workshops.

Although the IG showed statistically significant improvement in making food choices that are healthy for bones at week 8, this improvement was not sustained at 6 months, and it is unclear why. The 2015–2020 USDA dietary guidelines for healthy bones include promoting the consumption of dairy products, including whole and skim milk, at least 2 to 3 times/day (U.S. Department of Health and Human Services and U.S. Department of Agriculture, 2015). However, it is questionable if such guidelines are protective of bone health, and there are concerns that such recommendations may actually increase adverse health outcomes (Ganmaa et al., 2002; Ganmaa and Sato, 2005; Qin et al., 2009; Aune et al., 2015; Willett and Ludwig, 2020). It may be too much for some participants to consume large amounts of dairy products daily long term, especially for those who are lactose intolerant or who may not like the taste of milk. However, HESL does promote the consumption of other sources of calcium, including plant-based calcium sources such as tofu, nuts beans, broccoli, and kale (Messina and Mangels, 2001; American Dietetic and Dietitians of, 2003), as well as calcium supplements, to promote healthy bones.

Although participants were instructed on the health benefits of physical activity of various types including endurance, strength, flexibility, and balance exercises, during the intervention, we did not find a significant increase in the level of physical activity. We were informed by AgeSpan that the intervention did not focus on physical activity as much as it did on making dietary changes and that some participants opted out of the physical activity component.

WHR showed a significant decrease of 0.03 at month 6 in the IG compared to the CG (Table 5). WHR is a measure of central obesity or visceral fat, whereas BMI is a measure of overall obesity (Paniagua et al., 2008). Central obesity as measured using WHR has been shown to be a stronger predictor of chronic diseases, including heart disease, diabetes, and cancer, as well as mortality, than measures of total body fat such as BMI (Lapidus et al., 1984; Fujimoto et al., 1999; Janssen et al., 2002; Vazquez et al., 2007; Mathieu et al., 2009; Czernichow et al., 2011). The significant decrease in WHR at month 6 suggests that the healthy behavioral changes due to the HESL intervention may have a protective impact on an important risk factor of chronic diseases. In a study by de Koning et al. (2007), a 0.01 increase in WHR has been shown to be associated with a 5% increase in the risk of incident CVD events. It is not clear if the decrease in WHR in this study was due to changes in the WC and/or HC. However, a non-significant increase in HC was observed (Table 5).

Given that there was no follow-up of participants between week 8 and month 6, it is noteworthy that several significant improvements to healthy lifestyle behaviors observed at week 8, remained significant at month 6 (Table 4). It is difficult to predict if participants will continue to maintain the positive changes made to their lifestyle behaviors due to the intervention beyond the 6-month period. Future evaluations to determine factors associated with longer-term (i.e., beyond 6 months) effectiveness of HESL on healthy lifestyle behaviors, disease outcomes, and quality of life, among others, as well as to identify barriers to continuing adoption of these behaviors, are needed. We were not able to evaluate the impact of the intervention beyond 6 months due to a lack of resources. However, healthy lifestyle and behavioral changes can be maintained long term (Ornish et al., 1998; Ornish et al., 2005). It has been shown that those who made the greatest changes showed the biggest improvement (Ornish et al., 1998). In the BROAD study, overweight participants in the IG were empowered with knowledge of the benefits of a plant-based diet and encouraged to incorporate it into their lifestyles (Wright et al., 2017). The CG, also overweight participants, received standard medical care. Although the study lasted only 3 months, the plant-based group not only lost significant weight but also achieved greater weight loss at 6 and 12 months, derived physical and mental benefits, and stopped taking many of their medications, even though no further instructions were given beyond the 3 months intervention (Wright et al., 2017; Greger, 2020). Sustainable changes in dietary habits and behavioral lifestyles beyond the intervention period are possible when changes result in increased benefits and pleasure and/or are associated with positive emotions that naturally motivate the rewiring of the brain toward the adoption of such behaviors (Frates, 2016; Esselstyn, 2017; Fogg, 2020; Greger, 2020). Healthful habits repeated over time become a way of life (Fogg, 2020; Greger, 2020) and should be the ultimate goal of all interventions aimed at improving dietary and health behaviors. Although there was considerable variation across participants and behaviors (Gardner et al., 2012), it has been shown that automaticity plateaued on average around 66 days after the first daily performance (Phillippa et al., 2010). Working effortfully on a new behavior for 2 to 3 months may be appealing to participants interested in turning that behavior into a habit (Gardner et al., 2012).

Overall, findings in this work suggest that the HESL intervention positively impacts the healthy behaviors of its targeted population. However, several limitations should be acknowledged. These include a sample size of predominantly female and White, making the findings of this study likely not generalizable to males and persons of other ethnic groups. The present study used largely self-reported measures that were previously validated. Available resources did not allow for use of objective health measures (e.g., blood biomarkers), which would help further strengthen the study findings. Another limitation is missing pre-existing conditions and other data which may bias findings. For example, income was not included in the regression model. Although requested, only 21% of participants provided income data. The analyses, however, controlled for educational status, which serves as a surrogate variable for household income. As such, this bias is likely mitigated. Biases of randomization not being blinded were also limitations. The large loss to follow-up was another limitation of the study. While not receiving the intervention may be a reason for leaving the CG by approximately 23% of the CG participants, it is not clear why the intervention lost approximately 43% of its IG participants. Characteristics of participants in both groups who were lost to follow-up were not found to be significantly different when compared with those in these groups who remained in the study at week 8 and month 6 (data not shown). Future evaluations need to reach out to participants leaving the intervention to identify reasons for leaving, so future programs can remedy the situation.

The present study was able to explore the impact of the intervention based on self-reported measures. Although limited, findings suggest that the intervention has a positive impact on study participants’ health behaviors as well as a risk factor of chronic diseases (WHR), as reported by the participants themselves. Objective outcome measures that directly point to bone, heart, and overall health, are needed to support and strengthen current study findings. As such incorporation of such measures is needed in future evaluations of this intervention.

 A huge challenge in public health remains the need to minimize the large gap between existing evidenced-based knowledge on healthy diets and lifestyle behaviors and their actual adoption as a way of life by individuals in all age groups (Willett, 2019). Closing this gap requires multiple strategies. The HESL attempts to address this challenge in older adults through community education. The HESL program implementers will continue to adjust the program to optimize its effectiveness and its ability to change behaviors in the long term, beyond the intervention period, and to expand its reach to other states.

## Data Availability

The raw data supporting the conclusion of this article will be made available by the authors, without undue reservation.
