# Supplementary material for: Healthy Eating for Successful Living in Older Adults™ community education program—evaluation of lifestyle behaviors: A randomized controlled trial
Source: Front Aging. 2022 Sep 6;3:960986. doi: 10.3389/fragi.2022.960986 (PMC9521496; doi:10.3389/fragi.2022.960986)
Supplement: Supplementary file 1 [file DataSheet1.docx]

**SUPPLEMENTARY MATERIALS**

Supplementary Table 1. Comparison of Linear Random-Effects Model and the Logit

Mixed-Effects Model on Lifestyle Behaviors for All Participants

| **Evaluation Questions** | **Adjusted DID (linear)** | | **Adjusted DID (logit)** | |
| --- | --- | --- | --- | --- |
| **(Outcomes Measures)** | **Week 8** | **Month 6** | **Week 8** | **Month 6** |
| Made food choices for healthier bones (%) | 23.8*** | 7.4 | 1.88** | 0.46 |
| Made food choices for healthier heart (%) | 32.8*** | 31.6*** | 3.65*** | 3.554*** |
| Read nutrition labels when shopping/planning meals (%) | 12.3* | 21.7*** | 1.35 | 2.984** |
| Used MyPlate^TM^ tools for food choices (%) | 33.7*** | 31.1*** | 2.11** | 1.729* |
| Current smoker (%) | -3.2 | -1.4 | -1.73 | -0.62 |
| Sleep hours/day in past month |  |  |  |  |
| 6-8 hours (%) | -3.9 | 4.1 | -0.55 | 0.58 |
| Overall sleep quality in the past month |  |  |  |  |
| Fairly good and very good (%) | 9.5* | 9.5 | 1.73* | 1.79 |
| Confidence in managing most own health |  |  |  |  |
| Score 6 to 10 (%) | 16.7*** | 20.7*** | 2.11* | 3.031** |
| Found information provided by health care |  |  |  |  |
| providers useful /understandable |  |  |  |  |
| Score 6 to 10 (%) | 4.9 | 5.4 | 0.69 | 0.73 |
| Played active role in own health care and well-being |  |  |  |  |
| Score 6 to 10 (%) | 10.7** | 8.6* | 2.56* | 1.96 |

*p<0.05, **p<0.01, ***p<0.001

Data show beta-coefficients of outcome measures of interest. Both p-values and beta-coefficients were derived using linear random-effects model and the logistic mixed-effects model with binary outcome measures, adjusting for group (intervention=1, control=0); week 8 (week 8=1, 0=otherwise); month 6 (month 6=1, 0=otherwise); age (continuous variable); gender (male=1, female=0); education (college and above=1, 0=otherwise), marital status (married=1 and 0=otherwise), and race (White=1 and 0=otherwise).

| Supplementary Table 2. Intervention Impact on Lifestyle Behaviors (Participants Who Provided Data at All Three Timepoints) | | | | | | | | | | | | | | | | | | | |
| --- | --- | --- | --- | --- | --- | --- | --- | --- | --- | --- | --- | --- | --- | --- | --- | --- | --- | --- | --- |
| **Evaluation Questions**  **(Outcomes Measures)** | **Control** | | | | | | **Intervention** | | | | | | **Unadjusted DID ^α^** | | | **Adjusted DID ^β^** | | | |
|  | **Baseline** | | **Week 8** | | **Month 6** | | **Baseline** | | **Week 8** | | **Month 6** | | **Week 8** | **Month 6** | | | **Week 8** | | **Month 6** |
| Made food choices for healthier bones (%) | 40.0 | | 46.3 | | 50.7 | | 63.9 | | 78.3 | | 62.2 | | 8.1 | -12.4 | | | 16.1 | | -0.01 |
| Made food choices for healthier heart (%) | 44.8 | | 43.3 | | 47.8 | | 51.2 | | 80.4 | | 80.4 | | 30.7* | 26.2* | | | 40.1*** | | 36.6*** |
| Read nutrition labels when shopping/planning meals (%) | 33.3 | | 43.1 | | 43.1 | | 54.3 | | 76.1 | | 89.1 | | 12.0 | 25.1* | | | 15.6* | | 24.6*** |
| Used MyPlateTM tools for food choices (%) | 5.5 | | 12.3 | | 6.6 | | 13.8 | | 58.7 | | 45.7 | | 38.0*** | 30.8*** | | | 42.5*** | | 32.7*** |
| Current smoker (%) | 4.2 | | 5.6 | | 4.2 | | 6.5 | | 2.2 | | 4.3 | | -5.7 | -2.2 | | | -6.5* | | -2.4 |
| Sleep hours in past month |  | |  | |  | |  | |  | |  | |  |  | | |  | |  |
| 6-8 hours(%) | 84.7 | | 84.7 | | 81.9 | | 77.3 | | 71.7 | | 76.1 | | -5.5 | 1.6 | | | -7.1 | | 1.0 |
| Overall sleep quality in the past month |  | |  | |  | |  | |  | |  | |  |  | | |  | |  |
| Fairly good or very good (%) | 76.4 | | 74.6 | | 73.6 | | 80.0 | | 84.8 | | 84.8 | | 6.5 | 7.6 | | | 6.6 | | 6.6 |
| Confidence in managing health problems |  | |  | |  | |  | |  | |  | |  |  | | |  | |  |
| 6 to 10 (%) | 90.3 | | 93.0 | | 88.9 | | 73.3 | | 91.3 | | 87.0 | | 15.3 | 15 | | | 21.5** | | 20.9** |
| Found information provided by health care providers useful/understandable |  | |  | |  | |  | |  | |  | |  |  | | |  | |  |
| 6 to 10 (%) | 84.5 | | 91.5 | | 87.3 | | 82.2 | | 87.0 | | 87.0 | | -2.3 | 1.9 | | | 1.2 | | 1.9 |
| Played active role in own health care and well-being | |  | |  | |  | |  | |  | |  | |  |  | | |  | |
| 6 to 10 (%) | 87.5 | | 90.3 | | 93.1 | | 82.6 | | 97.8 | | 95.7 | | 12.4 | 7.5 | | | 11.5 | | 5.8 |
| Healthy Behavior Index (HBI) (Mean ± SD) | 5.5±1.7 | | 5.8±1.8 | | 5.7±1.8 | | 6.0±2.6 | | 7.2±1.7 | | 7.2±1.4 | | 0.9 | 1.0 | | | 1.4*** | | 1.3*** |
|  |  | |  | |  | |  | |  | |  | |  |  | | |  | |  |

*p<0.05, **p<0.01, ***p<0.001

Sample size varied by indicators. The sample size in the control group ranged from 55-72 and 41-46 in the intervention group.

**^α:^** Unadjusted DID (i.e., unadjusted intervention effect) denotes difference in difference, accounting for the baseline difference between the control and intervention groups. For example, the unadjusted DID result was calculated as ${(y}_{week 8}^{intervention}-y_{week 8}^{control})-{(y}_{baseline}^{intervention}-y_{baseline}^{control})$ for week 8, and positive and negative numbers under this column indicate the net increase or decrease in response (in percentage points) due to the intervention, respectively, at week 8; same at month 6 for all binary variables. For HBI, values are actual net increase or decrease due to the intervention. P -values were derived using a linear regression model.

**^β:^** Adjusted DID (i.e., adjusted intervention effect) and p-values derived from a random effects regression model that accounts for site clustering and repeated measures, with binary outcomes measures (1=healthy behavior, 0=otherwise; note exception: current smoker=1, 0=otherwise), adjusting for group (intervention=1, control=0); week 8 (week 8=1, 0=otherwise); month 6 (month 6=1, 0=otherwise); age (continuous variable); gender (male=1, female=0); education (college and above=1, 0=otherwise), marital status (married=1 and 0=otherwise), state (Massachusetts = 1 and 0 = otherwise), and race (White=1 and 0=otherwise). Positive and negative numbers under this column indicate the net increase or decrease in response (in percentage points) due to the intervention, respectively, at week 8; same at month 6 for all binary variables. For HBI, values are actual net increase or decrease due to the intervention.

Supplementary Table 3 Intervention Impact on Body Mass Index, Waist Circumference, Hip Circumference, Waist-to-Hip Circumference Ratio, Physical Activity, Social Connectedness, and Quality of Life (Participants Who Provided Data at All Three Timepoints)

| **Outcome Measures** | **Control (Mean ± SD)** | | | **Intervention (Mean ± SD)** | | | **Unadjusted DID ^α^** | | **Adjusted DID ^β^** | |
| --- | --- | --- | --- | --- | --- | --- | --- | --- | --- | --- |
|  | **Baseline** | **Week 8** | **Month 6** | **Baseline** | **Week 8** | **Month 6** | **Week 8** | **Month 6** | **Week 8** | **Month 6** |
| BMI Kg/m^2^ | 27.8±5.9 | 27.8±5.9 | 28.2±6.1 | 30.2±6.3 | 29.5±6.5 | 29.6±6.6 | -0.7 | -0.9 | -0.7 | 0.3 |
| Waist Circumference (inches) | 37.2±5.9 | 37.4±6.2 | 37.1±5.7 | 39.5±5.7 | 40.5±5.3 | 40.3±5.9 | 0.7 | 0.8 | -0.05 | 0.53 |
| Hip Circumference (inches) | 41.8±5.6 | 41.6±5.7 | 41.5±5.2 | 44.2±5.1 | 44.7±5.7 | 45.4±7.2 | 0.7 | 1.6 | -0.2 | 1.6* |
| Waist to Hip Circumference Ratio (WHR) | 0.89 ±0.08 | 0.90 ±0.08 | 0.89 ±0.07 | 0.89 ±0.08 | 0.91 ±0.08 | 0.90 ±0.09 | 0.01 | 0.00 | 0.01 | -0.01 |
| Physical Activity (MET-hrs/week) | 54.5± 50.3 | 56.0± 51.7 | 57.7± 48.4 | 58.8± 109.2 | 46.4± 83.3 | 42.8± 59.1 | -13.8 | -19.2 | -6.8 | -1.2 |
| Social Connectedness Score | 4.7±3.2 | 4.7±3.0 | 4.7±3.2 | 5.0 ±2.9 | 4.7 ±2.9 | 4.9 ±3.1 | -0.2 | 0.0 | -0.3 | -0.3 |
| Quality of Life | 81.2 ±13.0 | 81.8 ±11.8 | 80.7±12.0 | 79.1±14.5 | 82.4 ±10.8 | 79.6 ±18.0 | 2.8 | 1.0 | 2.2 | 1.0 |

*p<0.05 Sample size varied by indicators. The sample size in the control group ranged from 72-100 and 46-69 in the intervention group.

**^α:^** Unadjusted DID (i.e., unadjusted intervention effect) denotes difference in difference accounting for the baseline difference between the control and intervention groups. For example, the unadjusted DID result was calculated as ${(y}_{week 8}^{intervention}-y_{week 8}^{control})-{(y}_{baseline}^{intervention}-y_{baseline}^{control})$ for week 8, and positive and negative numbers under this column indicate the net increase or decrease in value due to the intervention, respectively at week 8; same at month 6. P -values were derived using a linear regression model.

**^β:^** Adjusted DID (i.e., adjusted intervention effect) and p-values derived from a random effects regression model that accounts for site clustering and repeated measures, with outcome measures as continuous variables, adjusting for Group (Intervention=1, Control=0); week 8 (week 8=1, 0=otherwise); month 6 (month 6=1, 0=otherwise); age (continuous variable); gender (male=1, female=0); education (college and above=1, 0=otherwise), marital status (married=1 and 0=otherwise), race (White=1 and 0=otherwise), state (Massachusetts = 1 and 0 = otherwise), and marital status (married=1, 0=otherwise). Positive and negative numbers under this column indicate the net increase or decrease in value due to the intervention, respectively at week 8; same at month 6.
